# Efficacy and cost‐effectiveness analysis of pretreatment percutaneous endoscopic gastrostomy in unresectable locally advanced esophageal cancer patients treated with concurrent chemoradiotherapy (GASTO 1059)

**DOI:** 10.1002/cam4.6136

**Published:** 2023-06-16

**Authors:** XinLei Ai, PengXin Zhang, XinMin Xie, Bo Qiu, YuJia Zhu, Lei Zhao, Mian Xi, YingJia Wu, SuPing Guo, JinYu Guo, FangJie Liu, DaQuan Wang, NaiBin Chen, QianWen He, YongHong Hu, MengZhong Liu, ZhaoXia Ding, Hui Liu

**Affiliations:** ^1^ Department of Radiation Oncology Sun Yat‐sen University Cancer Center Guangzhou P.R. China; ^2^ State Key Laboratory of Oncology in South China Sun Yat‐sen University Cancer Center Guangzhou P.R. China; ^3^ Collaborative Innovation Center for Cancer Medicine Sun Yat‐sen University Cancer Center Guangzhou P.R. China; ^4^ Lung Cancer Institute of Sun Yat‐sen University Guangzhou P.R. China; ^5^ Department of Financial Sun Yat‐sen University Cancer Center Guangzhou P.R. China; ^6^ Guangdong Association Study of Thoracic Oncology Guangzhou P.R. China; ^7^ School of Business Sun Yat‐sen Univeristy Guangzhou P.R. China

**Keywords:** concurrent chemoradiotherapy, cost‐effectiveness analysis, esophageal squamous cell carcinoma, percutaneous endoscopic gastrostomy

## Abstract

**Background:**

We launched a single‐arm phase II study to determine the efficacy and cost‐effectiveness of percutaneous endoscopic gastrostomy (PEG) before concurrent chemoradiotherapy (CCRT) in patients with esophageal squamous cell carcinoma (ESCC).

**Methods:**

Eligible patients received pretreatment PEG and enteral nutrition during CCRT. The primary outcome was the change of weight during CCRT. The secondary outcome included nutrition status, loco‐regional objective response rate (ORR), loco‐regional progression‐free survival (LRFS), overall survival (OS), and toxicities. A 3‐state Markov model was applied for cost‐effectiveness analysis. Eligible patients were matched and compared with those who had nasogastric tube feeding (NTF) or oral nutritional supplements (ONS).

**Results:**

Sixty‐three eligible patients received pretreatment PEG‐based CCRT. The mean change of weight during CCRT was −1.4% (standard deviation, 4.4%), and after CCRT, 28.6% of patients gained weight and 98.4% had normal albumin levels. The loco‐regional ORR and 1‐year LRFS were 98.4% and 88.3%. The incidence of grade ≥3 esophagitis was 14.3%. After matching, another 63 patients were included in the NTF group and 63 in the ONS group. More patients gained weight after CCRT in the PEG group (*p* = 0.001). The PEG group showed higher loco‐regional ORR (*p* = 0.036) and longer 1‐year LRFS (*p* = 0.030). In cost analysis, the PEG group showed an incremental cost‐effectiveness ratio of $3457.65 per quality‐adjusted life‐years (QALY) compared with the ONS group with a probability of cost‐effectiveness of 77.7% at the $10,000 per QALY willingness‐to‐pay threshold.

**Conclusion:**

Pretreatment PEG is associated with better nutritional status and treatment outcome in ESCC patients treated with CCRT compared with ONS and NTF. Pretreatment of PEG can be cost‐effective because of its significant clinical benefits.

## 1 INTRODUCTION

Esophageal squamous cell carcinoma (ESCC) is a common malignancy of the gastrointestinal tract in Asia.^1^ Among all types of cancer, patients with ESCC have a high risk of malnutrition, reaching 67% to 85%.^2^, ^3^ In patients with esophageal adenocarcinoma, obesity and overweight are risk factors and many patients still are overweight at the clinical presentation even with swallowing impairment. In contrast, our previous study found that ESCC patients had a high incidence of malnutrition.^4^ Malnutrition is related to the deterioration of the general condition, a worse response to treatment, increased adverse events, a prolonged hospitalization, which may further increase the economic burden of patients.^5^, ^6^ Patients with unresectable esophageal cancer usually have swallowing difficulties and severe esophagitis induced by concurrent chemoradiotherapy (CCRT). Previous studies suggested that percutaneous endoscopic gastrostomy (PEG) had a lower risk of procedure‐related morbidity and provided better nutritional support compared with nasogastric tube feeding (NTF).^7^ However, the timing of PEG feeding during CCRT in esophageal cancer has not been clearly established and was commonly started when patients had significant swallowing pain and continuous weight loss, which could lead to poor compliance to CCRT and adverse treatment outcome. On the other hand, some studies found that pretreatment PEG feeding led to limited weight loss, ensuring effective and safe nutritional status during CCRT.^8^, ^9^

The goal of nutritional support is either to correct existing malnutrition or to prevent nutrient depletion in patients who are normally nourished but metabolically stressed or anorexic.^10^ During CCRT, the commonly used enteral nutrition support for patients with esophageal cancer include oral nutritional supplements (ONS), NTF, and PEG. NTF and PEG usage has increased over the past decade because ONS could hardly meet the nutritional needs of patients with dysphagia. PEG has been reported to have the advantage of allowing higher energy feeds^11^ and shortening the length of hospital stay compared to nasogastric tubes.^12^, ^13^ PEG may be superior to the nasogastric tube in patients with radiation esophagitis and long‐term impaired swallowing function.^14^, ^15^ Due to the current high expenditure on healthcare, the efficacy and cost‐effectiveness of nutritional support need to be documented. Recently, value‐based care has attracted the attention of policymakers to control costs and maintain quality.^16^, ^17^ With the lack of an evidence‐based consensus on the preferred enteral nutrition support approaches, it is crucial to assess the relative value proposition of these nutritional support approaches with clinical data and costs.

We launched a single‐arm phase II study to determine the efficacy and cost‐effectiveness of PEG before CCRT in unresectable locally advanced ESCC patients by assessing their nutritional status and treatment outcome, then comparing the cost‐effectiveness in baseline matched among PEG, TNF, and ONS groups.

## 2 MATERIALS AND METHODS

### 2.1 Study design

This single‐arm phase II trial was conducted at Sun Yat‐sen University Cancer Center (Clinicaltrials.gov, Identifier NCT04380480). Study subjects were recruited from May 15, 2018 to March 19, 2020. Eligibility criteria included the following: (1) 18–75 years of age, (2) histologically proven esophageal squamous cell carcinoma, (3) inoperable stage II‐IVA disease based on the TNM staging system proposed by the Union for International Cancer Control (UICC) 2002, (4) Eastern Cooperative Oncology Group (ECOG) performance status (PS) 0–1, (5) Patient‐Generated Subjective Global Assessment (PG‐SGA) grade of A‐B, and (6) estimated life expectancy is at least 6 months. The main exclusion criteria included: (1) contraindications for percutaneous endoscopic gastrostomy; (2) contraindication for chemotherapy or radiotherapy; (3) severe gastrointestinal dysfunction or enteral nutrition intolerance and (4) severe vomiting, bowel obstruction, or gastrointestinal bleeding. This study was approved by the institutional review board. All patients signed a written informed consent form.

### 2.2 Procedures

Patients received percutaneous endoscopic gastrostomy 1–2 weeks prior to CCRT. Gastrostomy tubes were placed via the “pull” technique, the method initially introduced by Gauderer et al.^18^ Antibiotics and proton pump inhibitors were used for 3 days after PEG. All patients had access to the hospital diet. Supplements were prescribed to meet estimated nutritional needs (30–35 kcal/kg of energy, 1.2–1.5 g/kg of protein, and electrolyte supplementation each day). Feeding tubes will be retained for 1 month after CCRT.

Patients were immobilized according to the standard protocol in our center.^19^ The planning target volume (PTV), PTV‐gross tumor volume (GTV), and PTV‐clinical target volume (CTV) corresponded to GTV and CTV plus 0.5 cm, respectively. A dose of 60 Gy in 24 fractions was delivered to PTV‐GTV and 40 Gy in 16 fractions to PTV‐CTV. At least 95% of PTV received 95% of the prescription dose. Dose limitation for the organs at risk were as follows: lungs V20 ≤35%, mean lung dose ≤15 Gy, spinal cord ≤45 Gy, heart V30 <30%, kidney V10 ≤10%, liver V20 <20%. Daily cone‐beam CT (CBCT) was conducted for image guidance.

S‐1 was administered concurrently via the oral route twice a day for 40 mg/m^2^ from day 1 to day 14. This regimen was repeated every 3 weeks for three cycles. Dosage of S‐1 can be adjusted according to the treatment‐related toxicities. When concurrent chemotherapy was postponed for more than two consecutive weeks, chemotherapy should be discontinued.

Hematological tests and weight were evaluated weekly during treatment. Adverse events assessment was according to the Common Terminology Criteria for Adverse Events (version 5.0). The tumor response was evaluated 2 months after the CCRT based on endoscopy and biopsy (if necessary), contrast‐enhanced computed tomography (CT) of the neck, chest, and upper abdomen, and chest magnetic resonance imaging (MRI). After the tumor response evaluation, a percutaneous endoscopic gastrostomy tube could be removed for patients without deep ulcers or residual tumors in their esophagus. Patients then received regular follow‐ups thereafter.

### 2.3 Outcomes

The primary endpoint was the change of weight during CCRT, which was defined as the percentage of weight loss from baseline till the end of CCRT. The secondary endpoints were nutrition status, measured by the change of albumin, hemoglobin, and CRP level during CCRT; loco‐regional objective response rate (ORR) was evaluated by tumor in the esophagus and mediastinal nodes, excluding distant metastasis; loco‐regional progression‐free survival (LRFS), defined as the time from enrollment to the date of loco‐regional disease progression or death from any cause or censored at the last date of follow‐up; overall survival (OS), defined as the time from enrollment to death or censored at the last date of the follow‐up; ≥3‐grade acute esophagitis, pneumonia, and hematological toxicity. Nutrition status was evaluated at the end of CCRT. ORR was assessed 2 months after CCRT. Acute toxicities were evaluated from the start till 3 months after CCRT.

### 2.4 Baseline matched comparison among PEG, ONS, and NTF

**FIGURE 1:**
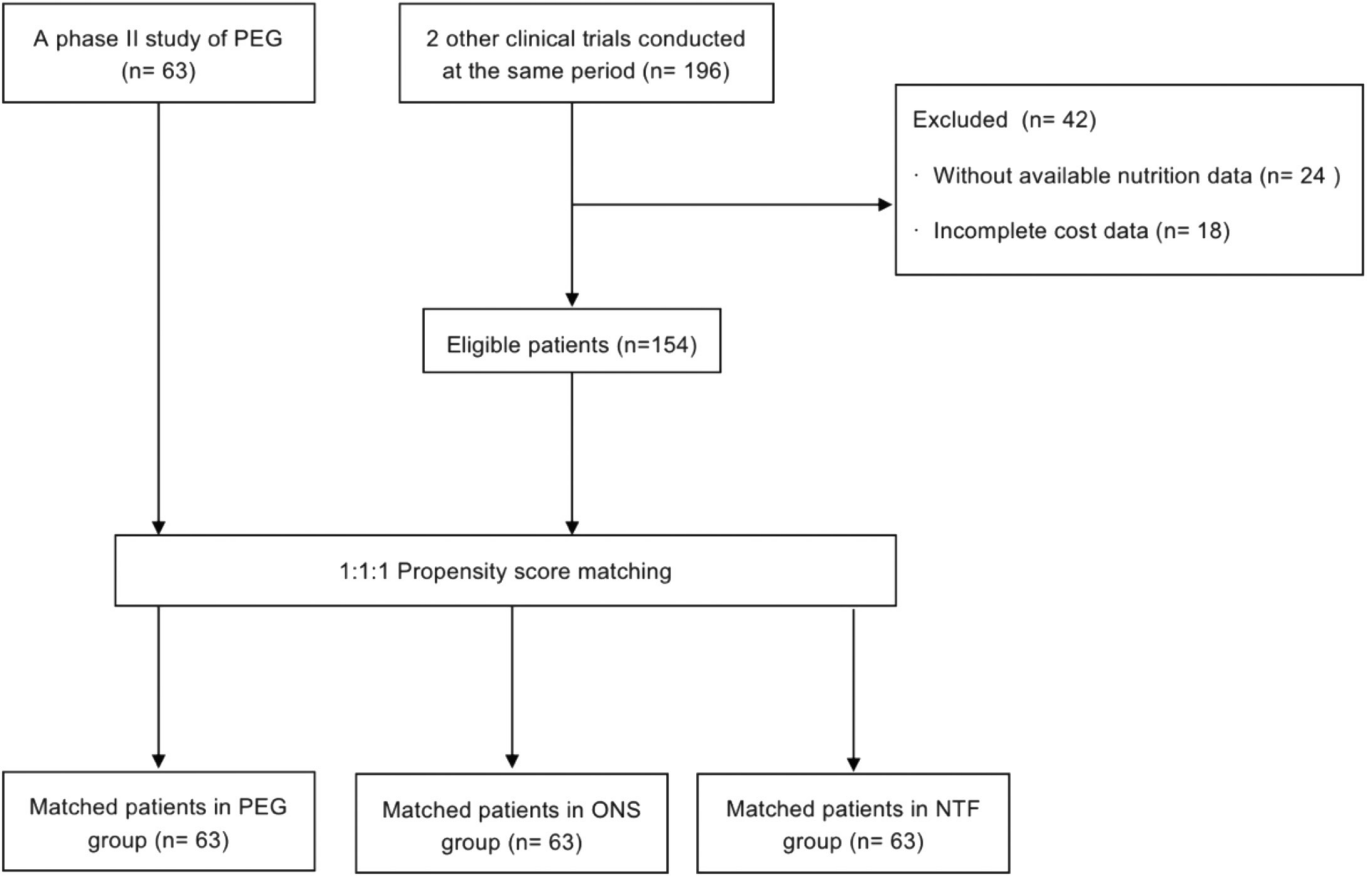
Flow diagram for this study. NTF, nasogastric tube feeding; ONS, oral nutritional supplements; PEG, percutaneous endoscopic gastrostomy.

Patients with oral nutrition support (ONS group) and nasogastric tube feeding (TNF group) were included in other two clinical trials conducted in our center during the same period (NCT02403531 and NCT02969473). The matching was based on 1:1:1 matching within a prespecified caliper width without replacement (Figure 1). The propensity score was estimated by regressing the treatment status on clinically relevant variables (i.e. patients' age, gender, tumor stage, and ECOG PS) by using a multinomial logistic regression model. Through nearest‐neighbor matching algorithms, we obtained subjects by matching the subjects on the logit of the propensity score with a caliper of 0.6 of the standard deviation of the logit of the propensity score.

For NTF and ONS groups, supplements were prescribed to meet estimated nutritional needs (30–35 kcal/kg of energy, 1.0–1.5 g/kg of protein, and electrolyte supplementation each day).

### 2.5 Cost analysis

**FIGURE 2:**
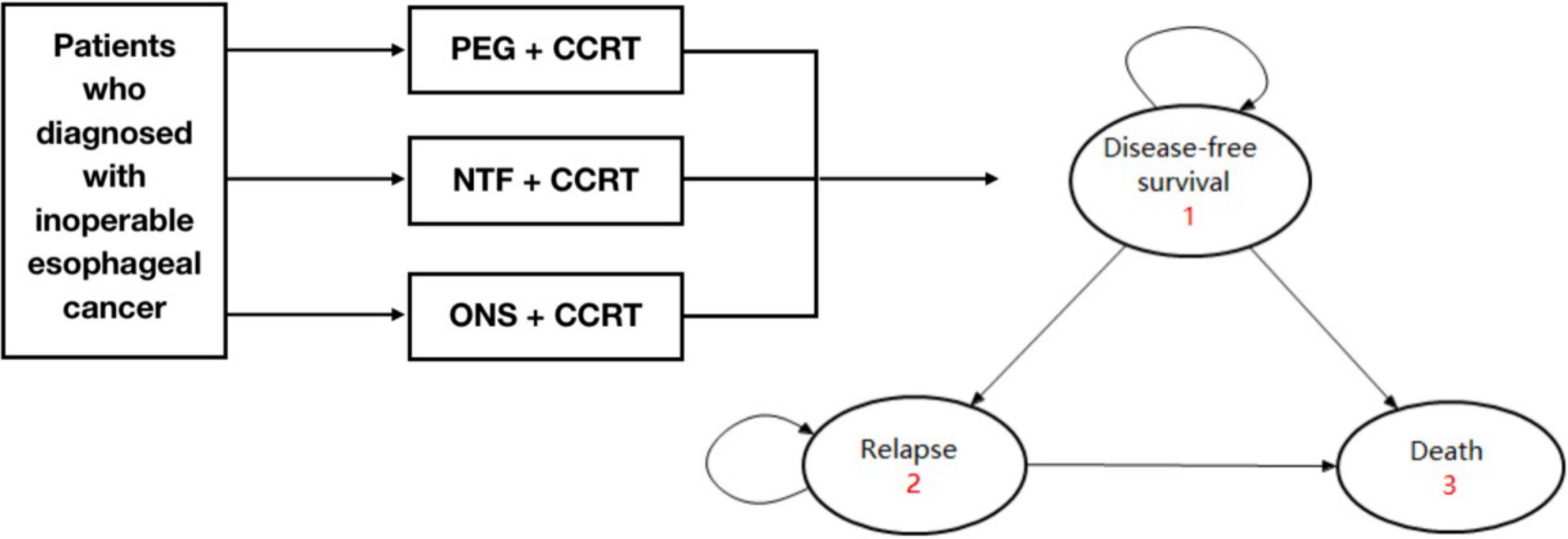
Diagram of transition states in Markov model. In the Markov model, 3 transition states of “disease‐free survival,” “relapse,” and “death” were used to simulate the disease process of the patient after radiotherapy. The model is structured over the time horizon of lifetime. 1‐year cycle length was used. CCRT, concurrent chemoradiotherapy; NTF, nasogastric tube feeding; ONS, oral nutritional supplements; PEG, percutaneous endoscopic gastrostomy.

A Markov‐based state transition model was established to simulate the clinical status of patients with inoperable ESCC (Figure 2). The baseline cohort included patients with a median age of 62 years treated with definite chemoradiotherapy for ESCC, using an annual cycle length. Key model parameters are presented in Table S1. All patients received either PEG or NTF or ONS and entered the model with free disease. During each cycle, the baseline cohort could remain stable or suffer disease relapse or death attributed to esophageal cancer or other causes. Risks of relapse and mortality following the three different enteral supports were derived from follow‐up data of patients included in this study. We adjusted survival to quality‐adjusted life years (QALYs) by using a utility value of 0.80 for a disease‐free state, 0.53 for a relapsed state, and 0 for death.

Cost included the following expenditure: radiotherapy, chemotherapy, nutrition support cost, treatment of major AEs costs, and hospitalization cost. Each price of these costs is based on our center. To compare the costs of different enteral nutrition supplement groups, healthcare costs during the initial 3 months and 6 months after the start of radiotherapy were analyzed. All costs were adjusted to US dollars with an exchange rate of $1 = ¥6.37 (December 7, 2021).

The cost‐effectiveness analysis followed the guidelines of the Second Panel on Cost‐Effectiveness in Health and Medicine.^20^ Incremental cost‐effectiveness ratio (ICER) was used to perform a comparative assessment, which is defined as the ratio of the difference in cost and QALYs between two possible strategies. Besides, all future costs and QALYs were discounted at 3% per year.

One‐way sensitivity analysis was performed to estimate the impact of different model parameters on the ICER. A probabilistic sensitivity analysis was performed to assess the uncertainty of the ICER estimate by using Monte Carlo simulation from 10,000 samples at different hypothetical willingness to pay (WTP) thresholds. The outcomes were reported as cost‐effectiveness probability for $10,000 per QALY and $50,000 per QALY as well as cost‐effectiveness acceptability curves. We applied international standards to define a strategy as “cost‐effective” if its ICER was below three times the gross domestic product (GDP) per capita in China (2020 annual: $33,962).^21^, ^22^

### 2.6 Statistical analysis

The primary endpoint of this trial was the change of weight during CCRT. We assumed that the change of weight could be reduced from −5.0% according to previous studies^23^ to −3% in the PEG group in the current study, with an estimated standard deviation of 5%. Therefore, the enrollment of 52 patients was required to yield 80% power to detect an expected reduction in weight loss on a one‐sided‐0.025 level test. Considering the rate of dropout as 15%, the planned enrollment was 62 patients.

A descriptive statistical analysis of baseline characterization and toxicity was performed. For all outcome measures, differences among groups were tested using one‐way ANOVA or Chi‐square test. Kaplan–Meier method was used to estimate the survival rates. We report the corresponding symmetric 95% confidence intervals (CIs). *p* value of 0.05 or less was considered statistically significant. Descriptive statistics and survival analysis were performed by SPSS 26.0. Propensity score analysis was performed using the SAS statistical software version 9.1 (SAS Institute). We construct a 3‐state Markov model to analyze the cost‐effectiveness using Treeage software (Treeage, Williamstown, MA, USA, 2019).

## 3 RESULTS

### 3.1 Patients' characteristics

From May 15, 2018 to March 19, 2020, 87 patients were assessed and 63 patients were enrolled in this study as the PEG group. Baseline characteristics are shown in Table 1. The median age of patients was 63 years (range 44–87). 56(88.9%) patients with stage III or IVA disease.

**TABLE 1:** Patients characteristics (*N* = 189).

	All groups (*N* = 189)	PEG group (*N* = 63)	NTF group (*N* = 63)	ONS group (*N* = 63)	*p* value
Median age, years (range)	62 (40–87)	63 (44–87)	60 (40–75)	63 (44–80)	0.119
Sex, No. (%)					
Male	152 (80.4)	47 (74.6)	54 (85.7)	50 (79.4)	0.296
Female	37 (19.6)	16 (25.4)	9 (14.3)	13 (20.6)	
Tumor stages, No. (%)					
IIA	8 (4.2)	2 (3.2)	2 (3.2)	4 (6.3)	0.101
IIB	8 (4.2)	5 (7.9)	0 (0)	3 (4.8)	
III	108 (57.1)	38 (60.3)	42 (66.7)	28 (44.4)	
IVA	60 (31.7)	18 (28.6)	17 (27.0)	25 (39.7)	
IVB	5 (2.6)	0 (0)	2 (3.2)	3 (4.8)	
Tumor location, No. (%)					
Cervical	21 (11.1)	5 (7.9)	7 (11.1)	9 (14.3)	0.378
Upper thoracic	63 (33.3)	20 (37.1)	21 (33.3)	22 (34.9)	
Middle thoracic	87 (46.0)	30 (47.6)	33 (52.5)	24 (38.1)	
Lower thoracic	13 (6.9)	7 (11.1)	1 (1.6)	5 (7.9)	
Synchronous multiple	5 (2.6)	1 (1.6)	1 (1.6)	3 (4.8)	
ECOG PS, No. (%)					
0–1	187 (98.9)	63 (100.0)	62 (98.4)	62 (98.4)	0.603
2	2 (1.1)	0 (0)	1 (1.6)	1 (1.6)	
Median weight (range)	58.4 (32.8–83.0)	58.7 (37.0–81.7)	58 (36.9–83.0)	58.4 (32.8–83.0)	0.813
Mean total radiation dose, Gy (range)	59.54 (30.00–69.92)	60.22 (57.50–65.00)	59.75 (30.00–67.40)	58.66 (44.00–69.92)	<0.001
Mean radiation fraction size, Gy (range)	2.23 (1.79–2.70)	2.49 (2.00–2.50)	2.10 (2.00–2.70)	2.09 (1.79–2.28)	<0.001
Overall treatment time, days (range)	36.1 (20–57)	33.0 (25–53)	37.8 (20–57)	37.5 (23–54)	<0.001

Abbreviations: ECOG PS, Eastern Cooperative Oncology Group Performance Status; NTF, nasogastric tube feeding; ONS, oral nutritional supplements; PEG, percutaneous endoscopic gastrostomy.

### 3.2 Treatment compliance

Of the 63 patients, 61 (96.8%) patients completed the full‐dose radiotherapy, and two patients failed to complete radiotherapy due to persisted grade of ≥2 esophagitis. Radiotherapy interruption occurred in 13 patients due to grade ≥3 esophagitis (*n* = 9), grade ≥3 hematological toxicity (*n* = 3), and heart failure (*n* = 1). Hence, 3 of 63 (4.8%) patients had a dose reduction of S‐1 due to hematological toxicities.

Of the 63 patients in the PEG group, 50 patients (79.4%) had gastrostomy feeding tube removal at the first or second follow‐up, and 5 patients (7.94%) retained their tube in the long term. The median duration of gastrostomy tube retention was 4.0 months (range, 2.9–33.73 months).

### 3.3 Nutrition evaluation

As shown in Table 2, the mean value of the change of weight was −1.4% (standard deviation, 4.4%) in the PEG group. Weight gain after CCRT occurred in 28.6% of the patients in the PEG group. However, 62 (98.4%) patients had ≥35 g/L albumin level after CCRT in the PEG group, 25(39.7%) patients had ≥15 mg/L CRP after CCRT in the PEG group, and 11 (17.5%) patients in the PEG group had LY# ≥1.0*10E9/L.

**TABLE 2:** Outcomes of three enteral nutrition support (*n* = 189).

	PEG group (*N* = 63)	NTF group (*N* = 63)	ONS group (*N* = 63)	*p* value
Tumor evaluation and survival				
Loco‐regional CR	50 (79.4%)	36 (57.1%)	34 (54.0%)	0.036
Loco‐regional PR	12 (19.0%)	21 (33.3%)	23 (36.5%)	
Loco‐regional SD	1 (1.6%)	2 (3.2%)	4 (6.3%)	
Loco‐regional PD	0 (0)	4 (6.3%)	2 (3.2%)	
Loco‐regional ORR	62 (98.4%)	50 (90.4%)	57 (90.5%)	
1‐year LRFS	88.3%	78.6%	76.8%	0.030
1‐year PFS	66.7%	63.0%	59.9%	0.185
1‐year OS	88.6%	83.1%	81.2%	0.576
Toxicities				
Grade ≥3 anemia, No. (%)	2 (3.2)	5 (7.9)	4 (6.3)	0.074
Grade ≥3 leukopenia, No. (%)	3 (4.8)	6 (9.5)	4 (6.3)	0.623
Grade ≥3 neutropenia, No. (%)	1 (1.6)	3 (4.8)	4 (6.3)	0.587
Grade ≥3 lymphopenia, No. (%)	58 (92.1)	56 (88.9)	55 (87.3)	0.485
Grade ≥3 thrombocytopenia, No. (%)	1 (1.6)	4 (6.3)	3 (4.8)	0.620
Grade ≥3 radiation esophagitis, No. (%)	9 (14.3)	19 (30.2)	20 (31.7)	0.045
Grade ≥3 radiation pneumonitis, No. (%)	0 (0)	1 (1.6)	0 (0)	0.145
Nutrition evaluation				
Weight change, percentage (mean ± standard deviation %)	−1.4 ± 4.4	−2.1 ± 3.3	−3.0 ± 5.4	0.134
Weight gain, No. (%)	18 (28.6)	8 (12.7)	7 (11.1)	0.001
Weight unchanged, No. (%)	7 (11.1)	26 (41.3)	21 (33.3)	
Weight loss, No. (%)	38 (60.3)	29 (46.0)	35 (55.6)	
Albumin level ≥35 g/L after CCRT, No. (%)	62 (98.4)	56 (88.9)	51 (81.0)	0.006
Albumin level <35 g/L after CCRT, No. (%)	1 (1.6)	7 (11.1)	12 (19.0)	
CRP level <15 mg/L after CCRT, No. (%)	38 (60.3)	27 (42.9)	25 (39.7)	0.044
CRP level ≥15 mg/L after CCRT, No. (%)	25 (39.7)	36 (57.1)	38 (60.3)	
LY# ≥1.0 × 10E9/L after CCRT, No. (%)	11 (17.5)	7 (11.1)	3 (4.8)	0.076
LY# <1.0 × 10E9/L after CCRT, No. (%)	52 (82.5)	56 (88.9)	60 (95.2)	
Treatment compliance and health status				
Completion for full‐dose radiotherapy, No. (%)	61 (96.8)	55 (87.3)	58 (92.1)	0.157
Radiotherapy interruption, No. (%)	13 (20.6)	30 (47.6)	25 (39.7)	0.005
Cost analysis				
Total cost from diagnosis till 6 months post‐CCRT ($)^a^	16,918.55	19,123.92	18,399.01	0.056
Length of hospital stay (days, during CCRT)	11.3	28.7	28.3	<0.001
Length of hospital stay (days, 6 months post‐CCRT)	2.9	2.8	3.1	0.918
Definitive radiotherapy cost ($)	10,086.04	8519.56	8377.22	<0.001
Concurrent chemotherapy cost ($)	695.95	1362.66	2248.82	0.001
Enteral nutrition support cost ($)	1120.28	732.56	757.65	0.001
Antibiotics and Emergencies cost ($)	332.98	1267.82	1073.23	<0.001

Abbreviations: CCRT, concurrent chemoradiotherapy; CR, complete response; NTF, nasogastric tube feeding; NTF, nasogastric tube feeding; ONS, oral nutritional supplements; ORR, overall response rate; PD, progressive disease; PEG, percutaneous endoscopic gastrostomy; PR, partial response; SD, stable disease.

^a^ $1 = ￥6.37 (December 7, 2021).

### 3.4 Tumor evaluation and survival

Two months after CCRT, there were 50 patients evaluated with loco‐regional complete remission (CR), and 12 with loco‐regional partial remission (PR) (Table 2). The loco‐regional ORR was 98.4% (62/63).

At the last follow‐up on April 9, 2021, the median follow‐up time was 18.7 (range, 7.8–31.7) months. The estimated 1‐year LRFS rate and 1‐year OS were 88.3% and 88.6%, respectively.

### 3.5 Toxicities

Common acute toxicities of patients treated with pretreatment PEG were also presented in Table 2. The incidence of grade ≥3 esophagitis was 14.3% (9/63). The most common grade ≥3 hematologic toxicity was lymphopenia (58/63, 92.1%), followed by leukopenia (3/63, 4.8%). Additionally, there were 3 patients experienced esophageal fistula, and 2 patients experienced esophageal hemorrhage. Six patients in the PEG group developed complications, one had a peristomal site infection (1.6%), three have tube blockage (4.8%), and two have tube dislodgement (3.2%).

### 3.6 Baseline matched analysis of PEG, NTF, and ONS groups

Process flow diagram of this study is shown in Figure 1. About 189 patients with stage II–IVA esophageal squamous carcinoma were enrolled, including 63 patients in the PEG group from NCT04380480 and 126 patients matched from 2 clinical trials (NCT02403531 and NCT02969473). The baseline characteristics of three groups did not differ statistically significantly from each other (Table 1). The PEG group had a higher mean total radiation dose (PEG group 60.22 Gy vs. NTF group 59.75 Gy vs. ONS group 58.66 Gy, *p* < 0.001) and higher radiation fraction size (PEG group 2.49 Gy vs. NTF group 2.10 Gy vs. ONS group 2.09 Gy, *p* < 0.001). The mean overall treatment time (OTT) was shorter in the PEG group (PEG group 33.0 days vs. NTF group 37.8 days vs. ONS group 37.5 days, *p* < 0.001).

During the whole treatment, more patients in the PEG group tended to complete the full‐dose radiotherapy (PEG group 96.8% vs. NTF: 87.3% vs. ONS 92.1%, *p* = 0.157). Thirty (47.6%) patients in the NTF group and 25 (39.7%) patients in the ONS group experienced radiotherapy interruption, while this occurred in 13 (20.6%) patients in the PEG group (*p* = 0.005).

As shown in Table 2, the mean change of weight was −1.4% (standard deviation, 4.4%) in the PEG group compared with −2.1% (standard deviation, 3.3%) in the NTF group (*p* = 0.435) and −3.0% (standard deviation, 5.4%) in the ONS group (*p* = 0.046). Weight gain after CCRT occurred in 28.6% of the patients in the PEG group, whereas 12.7% in the NTF group and 11.1% in the ONS group (*p* = 0.001). Approximately, 98.4% of patients had ≥35 g/L albumin level after CCRT in the PEG group, compared with 88.9% in the NTF group and 81.0% in the ONS group (*p* = 0.006), and 39.7% of patients had ≥15 mg/L CRP after CCRT in the PEG group, compared with 57.1% in the NTF group and 60.3% in the ONS group (*p* = 0.044). After CCRT, 11 (17.5%) patients in the PEG group had LY# ≥1.0*10E9/L, whereas only 7 (11.1%) in the NTF group and 3 (4.8%) in the ONS group (*p* = 0.076).

Four to eight weeks after the end of radiotherapy, the loco‐regional disease was evaluated for the groups (Table 2). Approximately, 79.4% of patients in the PEG group were found with complete remission (CR) compared with 57.1% in the NTF group, and 54.0% of patients in the ONS group. The loco‐regional ORR was higher in the PEG group compared with other groups (PEG group 98.4% vs. NTF group 90.4% vs. ONS group 90.5%, *p* = 0.036). As shown in Table S1, the 1‐year LRFS was 88.3% in the PEG group, 78.6% in the NTF group, and 76.8% in the ONS group (*p* = 0.030). The 1‐year OS was 88.6% in the PEG group, 83.1% in the NTF group, and 81.2% in the ONS group (*p* = 0.576).

Major treatment‐related toxicities in three groups were listed in Table 2. The incidences of grade ≥3 radiation esophagitis was significantly lower in the PEG group (PEG group: 14.3% vs. NTF group: 30.2% vs. ONS group: 31.7%, *p* = 0.045). The incidence of grade ≥3 anemia was higher in the NTF group (PEG group: 3.2% vs. NTF group: 7.9% vs. ONS group: 6.3%, *p* = 0.074). Incidence of grade ≥3 lymphopenia tended to be higher in the PEG group (PEG group: 92.1% vs. NTF group: 88.9% vs. ONS group: 87.3%, *p* = 0.485). No significant difference in other hematologic toxicities among the three groups was observed.

### 3.7 Cost‐effectiveness analysis

As detailed in Table 2, the average total costs from diagnosis till 6 months after CCRT were at $16,918.55 for the PEG group, $19,123.92 for the NTF group, and $18,399.01 for the ONS group (*p* = 0.056). The average medical costs of the radiotherapy were calculated at $10,086.04 for the PEG group, $8519.56 for the NTF group, and $8377.22 for the ONS group (*p* < 0.001). The enteral nutrition support cost was higher in the PEG group (*p* = 0.001). Total costs in 3 months and 6 months showed no statistically significant differences among the 3 enteral nutrition support groups. The PEG group had a significantly shorter length of hospital stay during CCRT (PEG group: 11.3 days vs. NTF group: 28.7 days vs. ONS group: 28.3 days, *p* < 0.001).

The 3‐state Markov model is shown in Figure 2. The results of the cost‐effectiveness analysis are presented in Table 3. By model calculation, the ICER associated with PEG compared with ONS was $3457.65 per QALY, which was much lower than the commonly accepted threshold for cost‐effectiveness ($33,962 per QALY in China). The probability that PEG was cost‐effective was 77.7% and 78.8% at WTP thresholds of $10,000 per QALY and $50,000 per QALY, respectively.

**TABLE 3:** Cost‐effectiveness analysis.

Strategy	Cost, $	QALYs	ICER ($/QALY)	Probability of cost‐effectiveness at $10,000/QALY, %	Probability of cost‐effectiveness at $50,000/QALY, %
ONS	27,753	4.53	–	5.7	5.8
NTF	28,691	5.06	1766.82	16.6	15.4
PEG	35,148	6.93	3457.65	77.7	78.8

Abbreviations: ICER, incremental cost‐effectiveness ratio; NTF, nasogastric tube feeding; ONS, oral nutrition support; PEG, percutaneous endoscopic gastrostomy; QALY, quality‐adjusted life‐year.

In one‐way analysis, the ICER was sensitive to the treatment cost of radiotherapy, routine follow‐up, and nutrition support cost and was also sensitive to the probability of mortality, relapse, and utility after treatment. The model variables in the order of their influence on the ICER were presented in Figure S1. In probabilistic sensitivity analysis, PEG was estimated to be the most cost‐effective strategy at the WTP thresholds of $5000 per QALY or higher (Figure S2).

## 4 DISCUSSION

Pretreatment PEG‐based CCRT showed promising treatment response and tolerable CCRT‐induced toxicities in unresectable locally advanced ESCC patients. The loco‐regional ORR and 1‐year LRFS were 98.4% and 88.3%. The incidence of grade ≥3 esophagitis was 14.3% (9/63) and no patient experienced grade ≥3 pneumonitis. In the current study, we also compared the efficacies, toxicities, nutrition status, and costs of three different enteral support groups. PEG group showed higher loco‐regional ORR (PEG group: 98.4% vs. NTF group: 90.4% vs. ONS group: 90.5%, *p* = 0.036), longer 1‐year LRFS (PEG group: 88.3% vs. NTF group: 78.6% and ONS group: 76.8%, *p* = 0.030). The PEG group had a significantly shorter length of hospital stay during CCRT (PEG group: 11.3 days vs. NTF group: 28.7 days vs. ONS group: 28.3 days, *p* < 0.001) and appeared to be cost‐effective due to significant survival benefits.

Our previous results found that the incidence of grade ≥3 radiation esophagitis including massive bleeding and perforation was significantly lower in ESCC patients with PEG (*p* = 0.032) when treated with definitive CCRT. The lower incidence of severe CCRT‐induced esophagitis in the PEG group suggested that adequate enteral nutrition promotes the repair of the esophageal mucosa. Tube feeding could also reduce the pain caused by oral feeding and the incidence of malnutrition. More patients in the PEG group gained weight after CCRT due to improved efficacy and reduced symptoms (PEG group: 28.6% vs. NTF group: 12.7% vs. ONS group: 11.1%, *p* = 0.001). In the current study, the daily energy and protein requirements of patients were 25–30 kcal/kg and 1.2–1.5 g of protein/kg, respectively. If severe protein depletion occurs, it is necessary to increase doses up to 2.0 g/kg/day to avoid the loss of lean body mass.^24^ Patients in the PEG group have better nutritional status and are mainly attributed to the following reason: 1. prepyloric tube feeding allows for large volumes and high osmotic loads, 2. NFT was generally retained for less than 4 weeks because of the associated discomfort and obstruction, and 3. the execution of oral feeding was less effective mainly because of the swallowing pain caused by CCRT‐related esophagitis.

Malnutrition predicts poor prognosis for esophageal cancer patients treated with chemotherapy or/and radiotherapy.^25^ According to the European Society for Clinical Nutrition and Metabolism guidelines on enteral nutrition for patients who receive CCRT, it is important to increase dietary intake and prevent treatment‐related weight loss, hematological adverse events, and treatment interruption.^25^ With respect to enteral nutrition supports during CCRT for esophageal patients, NTF and PEG groups have been shown to meet estimated nutritional needs according to the three important nutritional indicators including albumin, hemoglobin, and CRP.^26^, ^27^ In the PEG group, more patients had ≥35 g/L albumin level (PEG: 98.4%, NTF: 88.9%, ONS: 81.0%, *p* = 0.006) and had less incidence of grade ≥3 anemia (PEG: 3.2%, NTF: 7.9%, ONS: 6.3%, *p* = 0.074) during treatment. In addition, 11 (17.5%) patients in the PEG group had lymphocyte ≥1.0*10E9/L after CCRT, compared with 7 (11.1%) patients in the NTF group and 3 (4.8%) patients in the ONS group (*p* = 0.076). This group of patients is more likely to benefit from subsequent immunotherapy.

Six patients in the PEG group developed complications (*n* = 6, 9.5%), including peristomal site infection (*n* = 1, 1.6%), tube blockage (*n* = 3, 4.8%), and tube dislodgement (*n* = 2, 3.2%). The following measures should be taken to reduce the risk of PEG‐related complications^28^: (1) after PEG, patients will be given antibiotics (fluoroquinolones) for 3 days to prevent infection, (2) on the first day after the completion of PEG, the patient will be fasted from eating and drinking. Intravenous nutrition will be required according to the patient's weight and basal metabolism, (3) patients should mash the food into liquid and flush the tube with warm water after liquid food injection to prevent blockage, and (4) check balloon inflation volume at weekly intervals and inspect the water for evidence of stomach contents indicating balloon rupture.

We also found that the total costs of the PEG group from diagnosis till 6 months after CCRT were slightly lower among 3 groups (PEG group: $16,918.55 vs. NTF group: $19,123.92 vs. ONS group: $18,399.01, *p* = 0.056). As the costs of CCRT and enteral nutrition support were higher, the lower total treatment costs in the PEG group were probably related to the reduction in antibiotics and emergency treatment (PEG group $332.98 vs. NTF group $1267.82 vs. ONS group $1073.23, *p* < 0.001). Compared with the ONS group, NTF and PEG groups showed higher costs for nutrition support, which indicated more care for nutritional status. Besides, with the lower incidence of treatment‐related AEs and improved quality of enteral nutrition services, the length of hospitalization stay was reduced by half with 11.3 days in the PEG group. The shortened duration of CCRT not only contributed to the reduction of treatment costs but also minimized the length of hospitalization and long‐distance travel during the COVID‐19 epidemic. Moreover, the economic preferences for the PEG should be interpreted cautiously in the setting of different treatment approaches and economic environments.

To identify which treatment approaches are of the best value, we retrieved results from three prospective studies to compare the cost‐effectiveness of PEGs, NTFs, and ONS. We found that PEG is cost‐effective compared with NTF and ONS groups. Although ONS is more affordable, equally effective, and probably less toxic than NTF, we included NTF as a comparator because most patients with dysphagia are still undergoing NTF in China. Probabilistic sensitivity analysis showed that ONS is by no means to be a cost‐effective treatment unless the threshold of cost‐effectiveness analysis sharply fell to $5000 per QALY (Figure S2). But for now, it hardly seems possible that China's GDP fall to this level. To sum up, PEG is not only a safe and acceptable supportive approach but also improves the efficacy of CCRT and shows cost‐effectiveness benefits in esophageal cancer patients.

Our study has several potential limitations. First, our model‐derived short‐term costs related to the CCRT should not be extrapolated and was based on data from the observation period. However, it is inadequate for short‐term costs to represent the long‐term healthcare costs associated with living status. Second, the results of economic evaluations estimated for one country might not apply elsewhere. Further clinical trial compared these enteral nutrition supports was needed. Third, the concurrent chemotherapy regimen differs in three groups, with S1 in the PEG group and platinum‐based doublet regimen in NFT and ONS groups. Although previous trials using S‐1 as concurrent chemotherapy reported a comparable ORR rate and survival to those with DP‐ or PF‐based CCRT,^29^, ^30^, ^31^ the difference in chemotherapy intensity might be a confounder of the nutritional outcomes among the three groups. Further clinical trial directly comparing these enteral nutrition supports was needed.

## 5 CONCLUSIONS

Pretreatment PEG is a safe and effective approach for long‐term enteral nutrition in unresectable locally advanced ESCC patients treated with CCRT. The PEG group had better nutritional status, less incidence of severe esophagitis, and higher response to CCRT than the NTF and ONS groups. Compare with NTF and ONS groups, pretreatment of PEG can be cost‐effective because of significant clinical benefits.

## AUTHOR CONTRIBUTIONS

**XinLei Ai:** Data curation (lead); formal analysis (lead); resources (lead); software (lead); writing – original draft (lead). **PengXin Zhang:** Data curation (lead); formal analysis (lead); resources (lead); validation (equal); writing – original draft (lead); writing – review and editing (equal). **XinMin Xie:** Data curation (equal); formal analysis (equal); investigation (equal); project administration (equal); software (equal). **Bo Qiu:** Data curation (equal); investigation (equal); project administration (equal); supervision (equal); writing – review and editing (equal). **YuJia Zhu:** Methodology (equal); project administration (equal); resources (equal). **Lei Zhao:** Methodology (equal); project administration (equal); resources (equal). **Mian Xi:** Formal analysis (equal); project administration (equal); resources (equal). **YingJia Wu:** Funding acquisition (equal); project administration (equal); resources (equal). **SuPing Guo:** Project administration (equal); resources (equal). **JinYu Guo:** Project administration (equal). **FangJie Liu:** Project administration (equal); resources (equal). **DaQuan Wang:** Project administration (equal); resources (equal); software (equal). **NaiBin Chen:** Investigation (equal); project administration (equal); resources (equal); software (equal). **QianWen He:** Investigation (equal); project administration (equal). **YongHong Hu:** Investigation (equal). **MengZhong Liu:** Investigation (equal). **ZhaoXia Ding:** Conceptualization (equal); investigation (equal); methodology (equal); supervision (equal). **Hui Liu:** Conceptualization (lead); data curation (lead); methodology (lead); supervision (lead); writing – review and editing (lead).

## CONFLICT OF INTEREST

The authors declare that they have no known competing financial interests or personal relationships that could have appeared to influence the work reported in this paper.

## Supporting information


Figure S1.

## Data Availability

The datasets used and/or analyzed in the current study are available through the corresponding author on reasonable request.
