# Supplementary material for: Efficacy and cost‐effectiveness analysis of pretreatment percutaneous endoscopic gastrostomy in unresectable locally advanced esophageal cancer patients treated with concurrent chemoradiotherapy (GASTO 1059)
Source: Cancer Med. 2023 Jun 16;12(14):15000–10. doi: 10.1002/cam4.6136 (PMC10417071; doi:10.1002/cam4.6136)
Supplement: Supplementary file 1 — Figure S1. [file CAM4-12-15000-s001.docx]

Table S1. Model parameters of 3-state Markov model

| Model parameters | Values | Distributions | Sources |
| --- | --- | --- | --- |
| **Base case** |  |  |  |
| Age | 62 | N |  |
| **Model set up** |  |  |  |
| Cycle length | 1-year | N/A |  |
| No. of year^a^ | 15 | N/A |  |
| **Discount rate (annual)** | 3% | N/A |  |
| **Cost, $^b^** |  |  |  |
| Radiotherapy in PEG group | 10086.04 | γ | SYSUCC |
| Radiotherapy in NTF group | 8519.56 | γ | SYSUCC |
| Radiotherapy in ONS group | 8377.22 | γ | SYSUCC |
| Chemotherapy in PEG group | 695.95 | γ | SYSUCC |
| Chemotherapy in NTF group | 1362.66 | γ | SYSUCC |
| Chemotherapy in ONS group | 2248.82 | γ | SYSUCC |
| Nasogastric tube placement | 56.30 | N/A | SYSUCC |
| PEG tube placement | 823.63 | N/A | SYSUCC |
| Nutrition support in PEG group | 1120.28 | γ | SYSUCC |
| Nutrition support in NTF group | 732.56 | γ | SYSUCC |
| Nutrition support in ONS group | 757.65 | γ | SYSUCC |
| Hospital in PEG group | 48.57 | γ | SYSUCC |
| Hospital in NTF group | 123.36 | γ | SYSUCC |
| Hospital in ONS group | 121.64 | γ | SYSUCC |
| Antibiotics and emergencies in PEG group | 332.98 | γ | SYSUCC |
| Antibiotics and emergencies in NTF group | 1267.82  1895.77 | γ | SYSUCC |
| Antibiotics and emergencies in ONS group | 1073.23 | γ | SYSUCC |
| Routine disease free follow-up(annual) | 2038.72 | N/A | SYSUCC |
| First year of metastatic disease care | 9419.15 | γ | SYSUCC |
| Annual treatment of metastatic patients in remission | 2718.29 | N/A | SYSUCC |
| Utilities, QALYs |  |  |  |
| No cancer | 0.80 | β | Wildi et al.[20] |
| Alive with cancer | 0.53 | β | Wildi et al. [20] |
| Death | 0 | β |  |
| Rates and probabilities, % |  |  |  |
| Relapse after CCRT in PEG group(estimated annual rate) | 33.3 | β |  |
| Relapse after CCRT in NTF group(estimated annual rate) | 37.0 | β |  |
| Relapse after CCRT in ONS group(estimated annual rate) | 40.1 | β |  |
| Cancer deaths after CCRT in PEG group(estimated annual rate) | 11.4 | β |  |
| Cancer deaths after CCRT in NTF group(estimated annual rate) | 16.9 | β |  |
| Cancer deaths after CCRT in ONS group(estimated annual rate) | 18.8 | β |  |
| Risk of non-cancer death | 2018 Life Tables | N/A |  |

a. Markov models were to be cycled 15 times to evaluate the treatment outcomes over a 15-year time period for the base case until 77-year-old (Chinese life expectancy)

b. $ 1= ￥6.37 (2021.12.7)

Abbreviations: PEG= percutaneous endoscopic gastrostomy; NTF= nasogastric tube feeding ; ONS = oral nutrition support; QALY = quality-adjusted life-year；CCRT= concurrent chemoradiotherapy


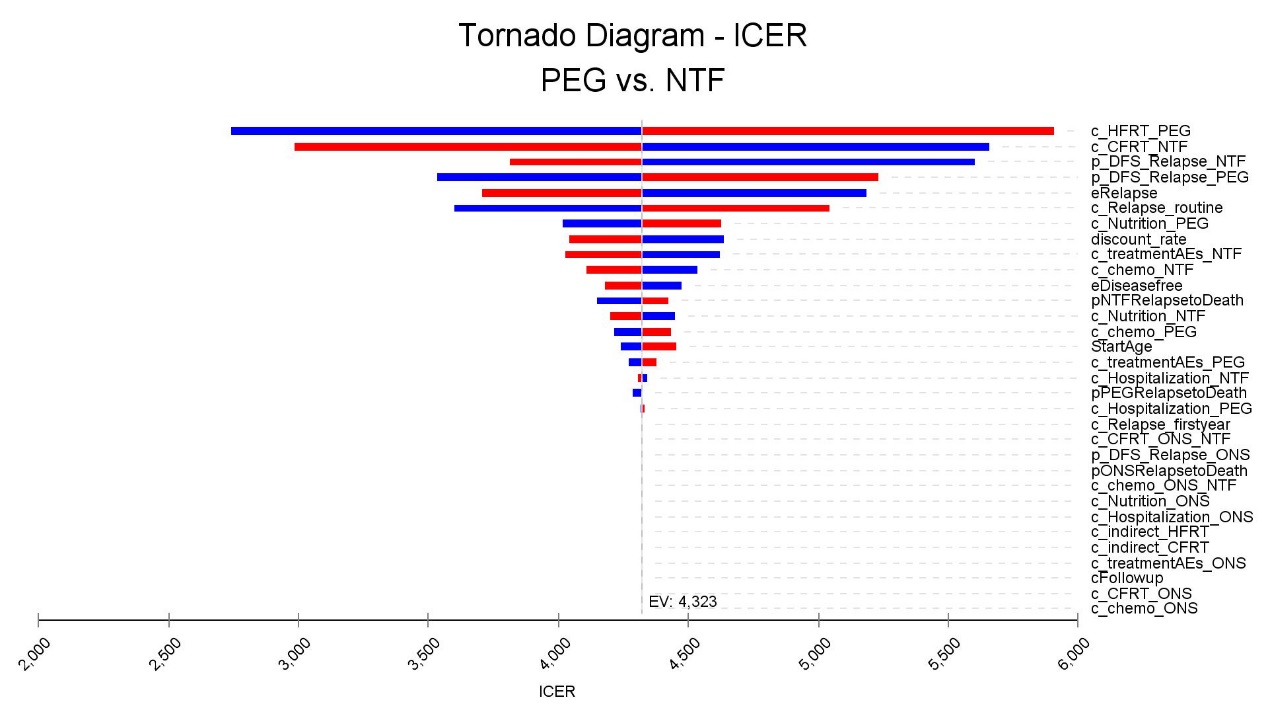


Figure S1. Tornado diagram of one-way sensitivity analysis for 3 groups.

Abbreviations: PEG= percutaneous endoscopic gastrostomy; NTF= nasogastric tube feeding; ICER = incremental cost-effectiveness ratio;

**A**


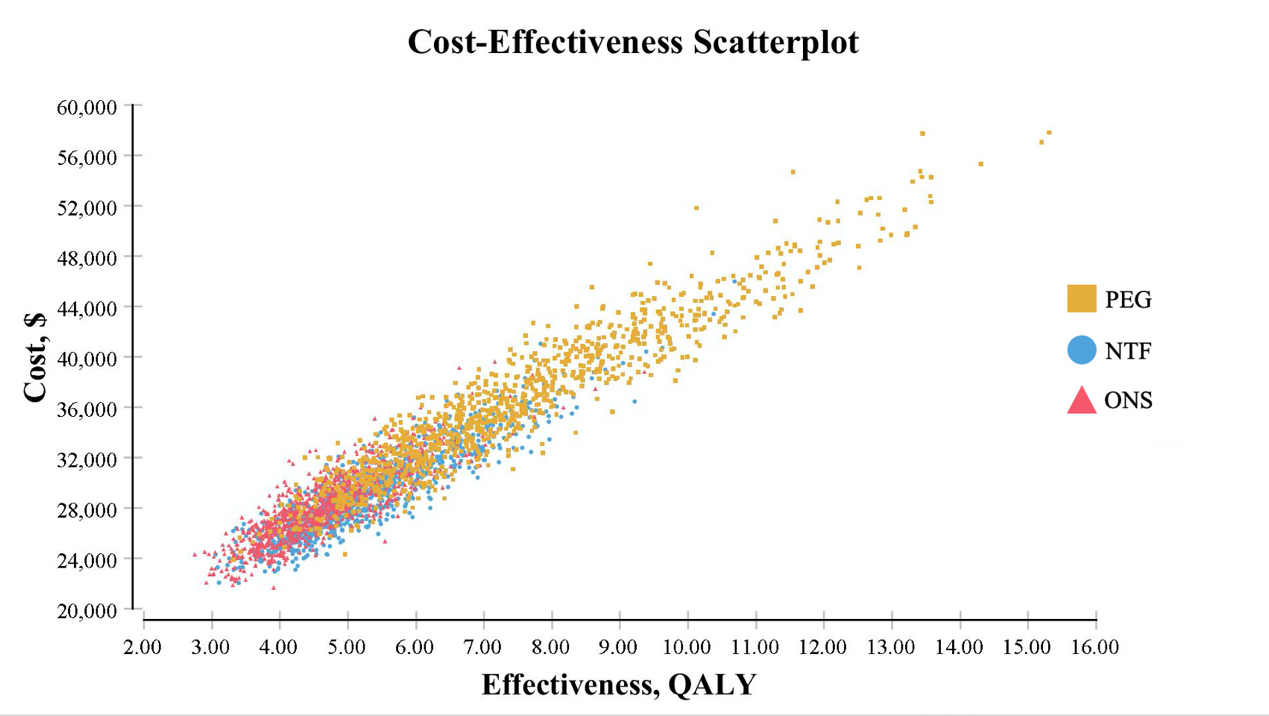


**B**


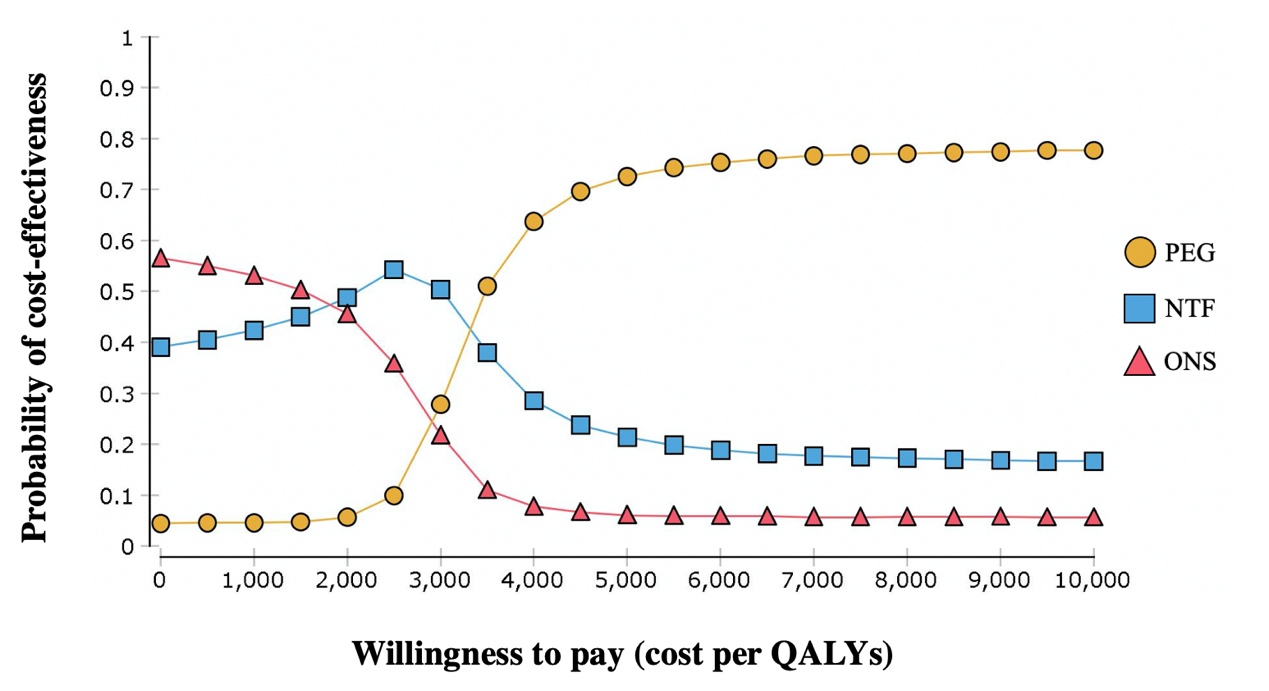


Figure S2. (A) Cost-effectiveness scatter plot of probabilistic sensitivity analysis. (B) Cost-effectiveness acceptability curve.

Abbreviations: CE = cost-effectiveness; PEG= percutaneous endoscopic gastrostomy; NTF= nasogastric tube feeding; ONS = oral nutrition support; ICER = incremental cost-effectiveness ratio;
